# Supplementary material for: Foot self-care practices among adults with diabetes in the West Bank, Palestine: A cross-sectional study
Source: PLOS Glob Public Health. 2026 Feb 20;6(2):e0005313. doi: 10.1371/journal.pgph.0005313 (PMC12922996; doi:10.1371/journal.pgph.0005313)
Supplement: Attachment — Submitted filename: point response additional.pdf [file pgph.0005313.s002.pdf]

Thank you for your message and for the in-house check. We have addressed each item as follows:

Data sharing restrictions / Data Availability Statement
a) Reason for restriction and who imposed it

The dataset contains human participant clinical and sociodemographic information. Although de-identification was performed, public sharing may still pose a risk of participant re-identification due to the combination of variables and the study context.

Accordingly, the dataset is not publicly available, consistent with ethical oversight requirements.

b) Data access mechanism and contact information:

De-identified data are available upon reasonable request to qualified researchers, subject to approval by the Institutional Review Board (IRB), Al-Quds University, and completion of a data-use agreement.

Data requests should be sent to: Research Office, Al-Quds University (Research@admin.alquds.edu).

We have updated the Data Availability statement in the Editorial Manager submission form to reflect the above.

Authorship change during revision

We have completed the Authorship Change Request form in full and uploaded it to the file inventory as Item Type "Other."

We have also uploaded a single PDF containing compiled email confirmations from all authors approving the authorship changes.

We trust these updates meet the journal's requirements. Please let us know if any additional information or formatting changes are needed.

Kind regards,
Dr. Salahaldeen Deeb
Corresponding Author
Al-Quds University
